# Perspectives and experiences of nurse academics teaching reflective practice to undergraduate Bachelor of Nursing students: a scoping review

**DOI:** 10.1177/17449871261445389

**Published:** 2026-10-15

**Authors:** Lisa Speedie, Gulzar Malik, Jacqueline Johnston, Christine East

**Affiliations:** Graduate Researcher, School of Nursing and Midwifery, La Trobe University, Bundoora, VIC, Australia; Associate Professor, School of Nursing and Midwifery, La Trobe University, Bundoora, VIC, Australia; Senior Lecturer, School of Nursing and Midwifery, La Trobe University, Bundoora, VIC, Australia; Professor, School of Nursing and Midwifery, La Trobe University, Bundoora, VIC, Australia

**Keywords:** academics, critical reflection, experiences, perspectives, reflective practice, undergraduate nurses

## Abstract

**Background:**

Reflective practice is essential in nursing education, as it promotes critical thinking and fosters professional growth. Although it is acknowledged in higher education and included in university attributes, the perspectives and experiences of nurse academics who teach it remain underexplored.

**Aim:**

This scoping review aimed to synthesise published primary studies exploring the perspectives and experiences of nurse academics teaching reflective practice to undergraduate Bachelor of Nursing students.

**Method:**

Databases searched included ProQuest Nursing, Medline, CINAHL, Scopus, Web of Science and the Cochrane Library, with a focus on primary studies from 2010 onwards. Using the Joanna Briggs Institute methodology and PRISMA guidelines, 11 studies were reported and thematically analysed.

**Results:**

From synthesising the included studies, four key themes were identified: (1) Reflective practice and the role of academics, (2) Reflective practice and academic challenges, (3) Reflective practice learning approaches and (4) Reflective practice and academic vulnerability.

**Conclusion:**

The studies revealed a limited understanding of the experiences and expectations of academics teaching reflective practice, with a focus primarily on identifying barriers rather than exploring the personal experiences of those who teach it. Further research is necessary to understand the perspectives and experiences of academics who teach reflective practice to undergraduate Bachelor of Nursing students.

## Introduction

Reflective practice is widely recognised as a vital skill for supporting critical thinking, clinical reasoning and professional development for Bachelor of Nursing students (Del Vecchio et al., 2024; Hallaran et al., 2023; Smith, 2021). Reflection not only improves practical skills and knowledge but also highlights the importance of reflection competencies (Jack and Illingworth, 2019) and strengthens critical thinking, self-awareness, empathy and thoughtful action (Graham and O’Brien, 2020; Zeng et al., 2024). Reflective practice is embedded in the Australian Nursing and Midwifery Board of Australia (NMBA, 2016) Registered Nurse Standards for Practice, and linked to professional growth and sustaining clinical competence. Nonetheless, both students and academics often encounter challenges in understanding and implementing reflective processes (Gathu, 2022).

Teaching reflective practice to undergraduate nursing students enrolled in a Bachelor’s programme, however, is a complex task that requires an in-depth understanding of pedagogical strategies to promote effective reflective practice (Aryuwat et al., 2024; Gustavsson et al., 2019). The growing recognition of the significance of reflection in nursing practice highlights the need for effective teaching methods that support students in acquiring this skill (Artioli et al., 2021; Legare and Armstrong, 2017). When academics lack the necessary training to model and facilitate reflective techniques, the quality of reflective learning decreases for both students and staff (Del Vecchio et al., 2024; Graham and O’Brien, 2020).

To maintain competence and adapt to the changing healthcare environment, academics must be equipped to practice and facilitate reflection (Naicker and Rensburg, 2018). Del Vecchio et al. (2024) stated that training and mentoring are necessary to enable effective reflection and to provide sufficient time to create a safe environment for academics and students to engage in reflection (Clarke, 2021; Kurosh et al., 2020; Lubbe and Botha, 2020).

### Significance of reflective practice in nursing education

Reflective practice facilitates the transition of nursing students from education to clinical practice and supports novice nurse educators in moving from clinical roles to academia (Legare and Armstrong, 2017). It helps students integrate theoretical knowledge with real-world experiences, fostering personal and professional growth (Schwind and Manankil-Rankin, 2020). Additionally, reflective practice contributes to the development of personal and aesthetic knowing, supporting holistic, person-centred relational practice (Horton et al., 2021; Schwind and Manankil-Rankin, 2020). Reflective practice also facilitates and supports novice nurse educators entering academia, promoting personal and professional development (Horton et al., 2021; Legare and Armstrong, 2017; Schwind and Manankil-Rankin, 2020).

To ensure competency and meet pedagogy requirements, academics must be equipped to practise and facilitate reflection, supported by adequate training, mentoring and time to create safe reflective spaces for staff and students (Clarke, 2021; Del Vecchio et al., 2024; Kurosh et al., 2020). Reflective practice extends beyond the evaluation of skills and the development of interpersonal understanding. It requires academics to model reflective thinking and encourage curiosity and critical questioning among students (Jacobs, 2016; Lubbe and Botha, 2020). Reflection is often understood as a process that occurs after an event, described by Schön (1983, 1987) as *reflection-on-action*. This concept has since been expanded to include *reflection-in-action*, which occurs during practice, and *reflection-before-action*, which involves anticipating and planning future practice (Schutz, 2026). Reflective practice typically involves analysing experiences to identify what worked well and what could be improved. Reflexivity, however, requires a deeper level of critical self-awareness (Sherwood, 2024). Reflexivity involves an ongoing internal dialogue through which academics examine their own agency, values and decision-making processes within their professional roles (Schutz, 2026). Reflexivity within individuals plays a role in generating knowledge, where personal experiences, beliefs and professional identities influence how knowledge is interpreted and constructed (Schutz, 2026; Sherwood, 2024). Through reflexivity, academics move beyond merely reviewing activities to critically examining how their beliefs and priorities shape their teaching practice and professional identity (Schutz, 2026).

Despite its acknowledged value, the literature on the perspectives and experiences of academics who teach reflective practice remains limited (Timizar-Le Pen et al., 2020). A lack of clarity or confidence in teaching reflective practice can hinder student development (Smadi et al., 2021). When academics are proficient in reflective practice, they not only enhance their professional growth but also contribute to curriculum development and improved student learning outcomes (Kurosh et al., 2020; Lubbe and Botha, 2020; Smadi et al., 2021). Recognising the importance of the academic experience is essential, particularly given that nursing curricula have embraced reflective practice as an effective tool for fostering self-awareness and developing critical thinking for over 20 years. However, responsibility has often rested with individual academics to apply reflective practice in ways that enhance and support student learning (Naicker and van Rensburg, 2018), a task that has posed several challenges and has often been poorly implemented or not implemented at all (Gustavsson et al., 2019).

Ossa Parra et al. (2015) argued that reflective skills can be taught but must be developed and practised over time (Chan and Lee, 2021; Gustavsson et al., 2019). A significant gap remains in the literature on developmental approaches to teaching reflective practice across higher education programmes, particularly in nursing (Smadi et al., 2021; Timizar-Le Pen et al., 2020). Academics need to understand their own beliefs and purpose when facilitating reflection; without this understanding, they limit both reflective learning and their students’ practices (Donohoe, 2019; Graham and O’Brien, 2020; Kurosh et al., 2020). However, without insights into the perspectives and experiences of those teaching reflection, the full impact of these dynamics on student learning remains unclear.

### Aim

This scoping review aims to synthesise the literature from primary studies that explore the perspectives and experiences of academics teaching reflective practice to undergraduate Bachelor of Nursing students. By examining existing literature, this review seeks to provide a comprehensive overview of the current state of knowledge in this area. The findings may inform future pedagogical strategies and contribute to the ongoing development of effective reflective practice in nursing education.

### Review question

What are the perspectives and experiences of academics teaching reflective practice to undergraduate Bachelor of Nursing students?

## Methods

The scoping review was conducted following the framework originally identified by Arksey and O’Malley (2005). The review followed the Joanna Briggs Institute (JBI) methodology, with the development of the protocol, inclusion criteria and the PRISMA (Figure 1) Extension for Scoping Reviews checklist for reporting and was registered with the Centre for Open Science Forum. This process provides a rigorous framework for systematically mapping, exploring and synthesising the evidence gathered (Arksey and O’Malley, 2005; Moola et al., 2020).

**Figure 1.**
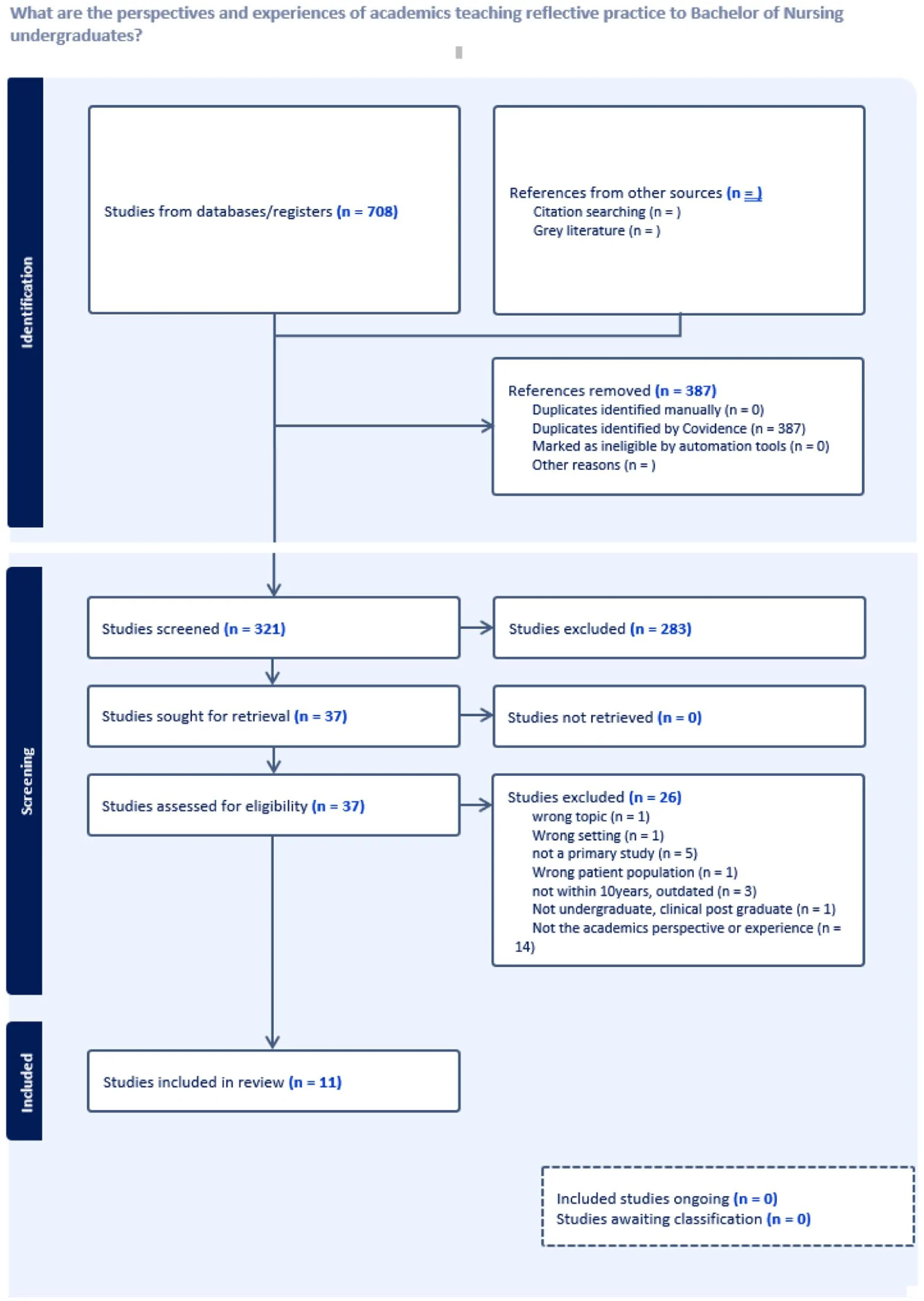
PRISMA Covidence.

### Search strategy

The initial search strategy was developed by the primary researcher and then refined collaboratively with the research team. Four key concepts and their synonyms (see Table 1) guided the search. Inclusion and exclusion criteria were developed using the PCC Framework (Population/Context/Concept) and are detailed in Table 2. An initial search was conducted across six electronic databases: ProQuest Nursing, Medline (Ovid), CINAHL, Scopus, Web of Science and the Cochrane Library. Manual searches of primary studies were also conducted at this time. Boolean operators and truncation symbols were used, and search strategies were tailored to each database (see Table 3 for examples).

**Table 1.** Concepts and terms.

Concept 1	Concept 2	Concept 3	Concept 4
Reflective terms	Where	Who by	What is required
Reflective practice Critical reflection Critical reflective practice Reflexivity Reflectivity	(Baccalaureate nurse terms for Bachelor of Nursing Course) Undergraduate courses Pre-registration course	Nurse teachers Nurse scholars Nurse academics Nurse facilitators Nurse academics	Skills Knowledge Experiences Perceptions Teaching Facilitation Attitudes

**Table 2.** Population, concept and context framework (PCC).

Inclusion criteria	Exclusion criteria
P – Patient, Population or Problem University academics teaching into nursing programmes/Bachelor of Nursing/Bachelor of Nursing Science – undergraduate nursing students, Bachelor Nursing Science students	Post graduate nurses, clinical practicing nurses, clinical academics/hospital facilitators
C: Context: Pre-registration bachelor of nursing; Undergraduate nursing programme	Teaching of reflective practice for practitioners only, for post-registration, post-registration clinical use only
C – concepts: reflective terms, reflective practice, academic skills/knowledge/perceptions/experiences, reflective tools in academia and curriculum	Master courses, post-registration courses, double degrees and graduate entry courses Non-English publications, publications before 2010

**Table 3.** Search strategy across the databases.

Data bases	Reflective practice terms	Additional terms
ProQuest – Nurs* AND (student OR undergrad OR college OR universit*)	‘reflective learning’ ‘Critical reflection’	Academic* Education*
CINAHL – ‘Education, Nursing, Baccalaureate+’) OR (MH ‘Students, Nursing+’) OR ‘Bachelor of nursing’ OR ((nurs* AND (undergrad* OR college* OR universit*))	CINAHL ‘Reflective Teaching practice*’ OR (teach* OR learn* OR educat*) N3 (‘reflective practice’ OR ‘reflective learning’ OR ‘critical reflection’))	Learn*
Medline – Bachelor of Nursing (nurs*and (undergrad* or college* or university))	((teach* or learn*) adj 3 (reflective practice or reflective learning or critical reflection))	3 and 4 teach* or learn* adj3

### Study selection process

The search was limited to publications from 2010 onwards, reflecting the introduction of reflective practice into undergraduate nurse education and the NMBA Registered Nurse Standards of practice from 2010. The inclusion criteria comprised primary research papers using qualitative, quantitative or mixed-methods designs, published in peer-reviewed journals.

Following the search, all identified citations were collated and uploaded into EndNote, and duplicates were removed. After removing duplicates, the search identified 321 potentially relevant studies [CINAHL (*n* = 154), Medline (*n* = 103), ProQuest (*n* = 27), Web of Science (*n* = 15) and Scopus (*n* = 12)]. All studies found through the manual search were identified in the databases. All 321 papers were imported into Covidence for screening. Titles and abstracts were independently reviewed by the lead researcher and two team members for suitability for the scoping review. At this stage, 281 studies were excluded. After initial screening, 38 full-text papers were reviewed by the lead and a second researcher for inclusion. Any conflicts were further reviewed and discussed within the team of three researchers to resolve. As a result, a further 27 studies were excluded, leaving 11 studies that met the inclusion criteria (see Figure 1).

### Quality appraisal

All 11 eligible studies were assessed for methodological quality using the JBI Quality Appraisal tool (Moola et al., 2020). Any conflicts were resolved by a third reviewer, as recommended by Pollock et al. (2023). All 11 studies were retained because the average score across the selected studies exceeded 65%.

### Data charting and extraction

For each paper, data were extracted, including the paper title, author(s), year, journal, country of publication, research purpose, research design, data collection methods, participants, key findings and quality appraisal score (see Table 4). The primary researcher extracted key data for each category and populated the table with information relevant to the review question. All researchers then reviewed the data to ensure consensus, and any conflicts were resolved through further discussion.

**Table 4.** Summary of findings.

Author/s and Year	Paper title	Journal	Country	Research purpose	Research design	Data collection	Participants/more detail	Key findings - expand	Quality appraisal score
Timizar-Le Pen et al. (2020)	Reflective writing: Implementation and learning perception from students and academics of French nursing schools.	*Nurse Education in Practice*	France	The study evaluates how nursing students write reflections on their professional experiences in nursing schools and examines the effects of these reflections on student learning.	A descriptive, multi-centred qualitative study.	Data collection involved two questionnaires: one for students and one for academics. They used four-point Likert scales with open-ended questions.	16 nursing schools, 5536 Semester 2 students and 266 academics were emailed the questionnaire. All 16 nursing schools participated, with 1,525 students (27.6% of 5,535) and 131 teachers (49.2% of 266).	This research explored the use of Situation Analysis (SA) in sixteen pre-registration French nursing schools as a structured method to help students develop reflective skills. Academics noted considerable variation in the delivery of SA. This study demonstrated that while academics valued SA for promoting reflexivity and professional development, they also noted challenges, including students’ limited understanding of reflection, inconsistent participation and the heavy workload involved in providing feedback and assessment. Suggested improvements included clearer objectives, stronger team cohesion, improved assessment processes and better support for both students and staff	7/8
Graham and O’Brien (2020)	Guided group reflection: A question of organisation and support for baccalaureate nursing students: Part 1	*Nurse Education in Practice*	UK, Ireland	This paper aims to explore the development of a guided reflective practice at an Irish university.	A case study was conducted to examine the implementation of guided reflective practice for fourth-year pre-registration nursing and midwifery students in Ireland	Data collection was integrated into the structured guided reflection process for final-year pre-registration of BSc Nursing and Midwifery students	The initiative involved final-year pre-registration BSc Nursing and Midwifery students in Ireland. For this study, groups included approximately 11 students each	The study revealed that providing guided, protected reflective time for fourth-year pre-registration nursing and midwifery students is resource-intensive but essential for easing their transition into professional practice. Overall, this initiative highlighted the importance of structured, collaborative and well-resourced strategies for integrating reflection into undergraduate nursing education	6/8
Gustavsson et al. (2019)	To challenge and to be challenged – academics collective learning in higher education	*Reflective Practice, International and Multidisciplinary Perspectives*	Sweden	This study aims to examine the characteristics of the academic role and the challenges faced in leading reflective seminars in higher education	The study was conducted within a three-year bachelor’s nursing programme at a Swedish university. In this university Reflective Supervision in Nursing (RHiO) seminars were introduced to encourage reflection and professional growth	Independent researchers conducted two group interviews. The interviews began with open-ended questions about RHiO, followed by discussions on academics’ experiences as supervisors, their perspectives on students’ reflective and professional identity development, and their own growth as supervisors	The study involved both students and academics in a three-year bachelor’s programme in nursing at a Swedish university. A total of 27 academics serving as Reflective Supervision in Nursing (RHiO) were invited to participate; 11 (nine women and two men) were aged 40–60. Of the student participants, 25 (from a total cohort of 5,535; 27.6% participation) contributed to the study	The study highlighted four key themes in the academic role during reflective seminars: (1) managing control and uncertainty, where academics balance responsibility for individual students and the group while allowing student narratives to shape content; (2) building trust while challenging assumptions to promote critical reflection; (3) shifting between closeness and distance, using students’ stories as a basis for reflection while maintaining perspective to support both professional and personal development; and (4) experiencing parallel processes of supervising and learning, with academics guiding student reflection while also enhancing their own pedagogical skills through collegial exchange. The study concludes that supervising reflective seminars requires university pedagogical expertise that differs from that required for other teaching formats, particularly in the capacity to connect theory with practice in real time and to guide reflection that challenges existing norms and professional cultures	5/8
Jack and Illingworth (2019)	Developing reflective thinking through Poetry Writing: Views from Students and Academics	*International Journal of Nursing Education Scholarship*	UK	This study explored students’ and academics’ perspectives on using poetry writing to enhance reflective engagement, rather than traditional models	An exploratory descriptive design was used. Thematic analysis was performed.	Data were collected via face-to-face, unstructured interviews with experienced academics who supervise RHiO seminars	The sample comprised 12 participants, including three nurse lecturers and nine nursing students, from the BSc (Hons) Adult Nursing programme	The study highlights four themes: *Less Restrictive than Reflective Models* Academics observed that poetry enabled students to transcend rigid structures and engage in more genuine, personal reflection on clinical practice. *Freedom of Expression* The academic saw poetry as a safe ‘space’ between the student and their experience, allowing for deeper reflection without the discomfort of speaking directly about themselves. *Development of Skills and Attitudes* Academics explained that poetry writing helps develop key skills and attitudes for nursing. *The Need for Careful Facilitation* Academics highlighted the importance of preparation, maintaining a non-judgemental attitude and respecting all contributions to create a supportive learning environment	5/8
Naicker and van Rensburg (2018)	Facilitation of Reflective Learning in Nursing: Reflective Teaching Practices of Academics	*African Journal of Nursing and Midwifery*	South Africa	The purpose of this study was to explore and describe the role of academics in facilitating students’ reflective learning	A quantitative, exploratory, descriptive approach was employed to investigate academics’ practices and perspectives on their role in facilitating reflective learning, as well as the appropriateness of the learning environment. The research was conducted at nine nursing education institutions in one South African province	Data were gathered through a structured self-report questionnaire. The tool consisted of three sections, featuring closed- and open-ended questions that allowed quantitative and qualitative responses.	The study population consisted of nurse academics who held an additional qualification in education, were registered with the professional body, and were employed at nursing education institutions (NEIs). Of the 231 questionnaires distributed, 121 were returned, resulting in a 52% response rate	**T**he study highlighted the following three themes: *Academics’ Own Reflective Practices* Many academics lacked formal training in reflective learning: only 46% reported studying it during their educator training, and 63% did not incorporate it into their own studies. *Facilitation of Reflective Learning* Despite limited personal experience, 98% of academics reported integrating reflective learning into their teaching. *Perceived Value and Learning Environment* Almost all respondents agreed that reflective learning benefits students (98%) and that their own reflective practice enhances teaching effectiveness (94%)	6/8
Koldestam et al. (2021)	Model for Improvements in Learning Outcomes (MILO): Development of a conceptual model grounded in caritative caring aimed to facilitate undergraduate nursing students’ learning during clinical practice (Part 1)	*Nurse Education in Practice*	Sweden	This study aims to describe the development of a caring conceptual model designed to enhance undergraduate nursing students’ learning during clinical practice	An exploratory design was used, combining a systematic literature review with the Delphi method to develop the conceptual model	Data collection involved a four-round Delphi process with 12 expert panellists who provided ongoing feedback based on their specialised knowledge during model development	The study involved a Delphi panel of 12 expert registered nurses from university and healthcare faculties in southern Sweden.	*Key Findings were*: The study developed the MILO, which includes eight interconnected core concepts, four intrapersonal and four contextual, designed to enhance undergraduate nursing students’ learning during clinical practice. Intrapersonal Concepts Academics identified nursing knowledge, a reflective approach, a critical approach and attention to quality and safety as fundamental characteristics and skills students need to develop. Contextual Concepts Environmental support is also considered essential. Peer learning, co-clinical teachers (from both university and healthcare faculties), student-centred and student-active supervision, and a supportive learning environment create the necessary framework for reflection and skill development. Overall Model: MILO supports students’ professional growth, facilitates educators’ application of a structured pedagogical approach and improves patient care	7/8
Lindberg (2018)	Lecturers’ lived experiences of guiding reflective seminars during nursing education	*Nurse Education in Practice*	Sweden	The study aimed to address the lack of pedagogical guidance for academics in facilitating reflective learning activities in nursing education	The study was guided by Reflective Lifeworld Research (RLR), a phenomenological approach .	Data were collected through in-depth interviews with academics experienced in leading reflective seminars in nursing education.	Eight academics (six women, two men) with experience leading reflective seminars in nursing education were interviewed	Key findings from the study are as follows: *A Foundation for Caring Science Builds Confidence.* Academics felt their caring science backgrounds and nursing experience strengthened their confidence in leading reflective seminars, but some noted that their personal assumptions could limit the openness of the students’ lived experiences. *Guiding Reflection Requires Ongoing Vigilance* Leading reflective seminars required constant focus, sensitivity and careful professional boundaries by academics. *A Lack of Trust in Oneself Inhibits Guiding Reflection.* Academics often questioned their ability to lead seminars effectively, especially when outcomes were uncertain or professional standards of expertise were challenged. *Closeness to Students* Reflective seminars created emotional closeness, leading to meaningful sharing, but this required academics to manage their own reactions and resilience. Overall, from the academics’ perspective, guiding reflective seminars requires deep personal involvement, pedagogical sensitivity and emotional resilience, often without formal preparation or institutional support	6/8
Mulli et al. (2022)	Undergraduate nursing simulation facilitators lived experience of facilitating reflection-in-action during high-fidelity simulation: A phenomenological study	*Nurse Education Today*	Canada	This study aimed to explore and describe the lived experiences of undergraduate nursing simulation academics who used reflection-in-action during high-fidelity simulations	A descriptive phenomenological design was used	Data were collected through semi-structured interviews following Bevan’s (2014) phenomenological interviewing method. Data were analysed using Colaizzi’s (1978) seven-step process	The study recruited simulation facilitators from eight colleges and universities across a Western Canadian province. Eligible participants needed to use high-fidelity simulation in undergraduate nursing programmes within academic settings. A total of 11 participants, all female and aged 36 to over 66 years, participated	Analysis identified four major themes and 14 subthemes, highlighting academics’ perspectives on reflection-in-action during high-fidelity simulation. *Essence of the Experience* Academics could recognise reflection-in-action by observing learner behaviours such as pausing, collaborating, verbalising, or adjusting actions. While they receive formal training in pre-briefing and debriefing, there is no structured education for reflection-in-action; instead, academics acquire this knowledge informally. Despite challenges, academics have devised strategies to facilitate reflection-in-action, considering it crucial for building confidence, critical thinking and reflective nursing practice	8/8
Smadi et al. (2021)	A Community of Inquiry lens into nursing education: The academics’ experiences and perspectives from three Australian universities	*Nurse Education in Practice*	Australia	The purpose of this study is to examine how Australian nurse academics design and deliver online and blended courses through the lens of the Community of Inquiry (CoI) framework	This study is qualitative, employing an explanatory sequential mixed-methods design	*Data Collection* Eleven semi-structured interviews Data Analysis Analysed using deductive thematic analysis (Braun & Clarke, 2022)	Twelve universities across six Australian states and territories were invited to participate, resulting in the purposive selection of 11 nurse academics from three universities for semi-structured interviews	The analysis identified three themes: cognitive, social and teaching presence: 1. *Cognitive Presence* Academics promoted reflection and inquiry via problem-based assignments, discussion forums and case studies 2. *Social Presence* Achieving social presence in the online learning environment was challenging due to isolation and distance. 3. *Teaching Presence* Nurse academics are responsible for designing and organising courses. The findings demonstrate that elements of the CoI framework are already embedded in Australian nursing education, although they are not always clearly articulated. The study highlights the need for more training, workshops and mentoring in CoI application to strengthen social and teaching presence, improve course design and provide quality online and blended learning experiences	7/8
Smith (2021)	Guided reflective writing as a teaching strategy to develop nursing student clinical judgement	*Nursing Forum*	United States	This qualitative descriptive study	A qualitative descriptive design was used to examine undergraduate nursing students’ and clinical faculty’s perspectives on guided reflective writing for developing clinical judgement	*Data collection* Data were gathered from both junior and senior pre-licensure BSN students through a 13-item open-ended survey after clinical experiences. Faculty insights were gathered in a focus group. Data analysis Survey and focus group data were analysed separately using Graneheim and Lundman’s content analysis method	The study was carried out at a small Midwestern university with a BSN programme of approximately 100 students. Participants included 47 nursing students (28 Junior One and 19 Senior Two). Four full-time academic members participated in a focus group	Analysis of student and faculty perspectives revealed four main subthemes: approach to thinking, application to clinical care, assignment guidance and assignment value, which shaped the development of group-specific themes. *Junior One students* emphasised that guided reflective writing helped them organise fundamental nursing care, supported structured reflection and prepared them for upcoming clinical experiences. *Senior two students* noted a sense of completeness, using the assignment to combine patient care priorities, connect different experiences and engage in deeper reflective thinking. *From an academic perspective*, the assignment's primary aim was to promote deep thinking. Faculty saw the tool as a meaningful guide for reflection, enhancing the development of clinical judgement while encouraging valuable interaction between students and academics	8/8
Jacobs (2016)	Reflective learning, reflective practice	*Nursing*	Canada	This paper aims to explore the meaning, importance and practice of self-reflection in nursing practice and education	Conceptual and reflective discussion paper, informed by literature and the author’s classroom teaching experience	Draws on educational literature, established reflective models	This is not an empirical study; perspectives are based on the author’s teaching practice with nursing students’ pre-registration and existing literature	This paper highlights the multifaceted role of reflective practice in nursing education and its vital contribution to the development of competent, compassionate and critically reflective practitioners. Overall, the findings highlight that reflective practice must go beyond superficial exercises and be genuinely modelled by nurse academics to effectively promote critical thinking, professional growth and holistic nursing practice	5/8

### Data analysis

The included studies were analysed using thematic analysis informed by Braun and Clarke (2022) and guided by an inductive content approach. This method aligns with thematic analysis, as categories were developed by comparing similarities and differences within the data (Kyngäs et al., 2020). The primary researcher immersed themselves in the data and identified the characteristics of each study. This mirrors thematic analysis, in which patterns and themes are identified and refined to ensure that the research question is addressed (Braun and Clarke, 2022; Munn et al., 2018). Themes were identified, and all researchers discussed them to review and verify them, including searching, categorising, defining and reviewing them again (Clarke and Braun, 2016; Kyngäs et al., 2020).

## Results

### Study characteristics

#### Search outcomes

Study characteristics from the 11 included studies are as follows: countries of origin were the United Kingdom (*n* = 3), Sweden (*n* = 2), Canada (*n* = 2), France (*n* = 1) and one study each from South Africa, the United States and Australia. Study designs included qualitative (*n* = 8), mixed methods (*n* = 2) and one quantitative study. Data collection techniques varied across and within the studies. The most frequently used were questionnaires/surveys (*n* = 6), in-person interviews (*n* = 3), focus groups (*n* = 3), which were used alongside interviews on two occasions, case studies (*n* = 2) and databases (*n* = 3).

Participants in the 11 studies comprised academics teaching in undergraduate Bachelor of Nursing programmes and students enrolled in these programmes. Among the included studies, Timizar-Le Pen et al. (2020) surveyed 16 nursing schools, whereas Gustavsson et al. (2019) involved 525 students and 131 academics across 16 schools. Jack and Illingworth (2019) engaged 12 participants (three academics and nine pre-registration students), and Smith (2021) gathered perspectives from both students and academics within a single programme. The remaining studies focused solely on academics: Naicker and van Rensburg (2018) (*n* = 121), Graham and O’Brien (2020) (11 academics/facilitators), Koldestam et al. (2021) (12 Delphi panel academics), Lindberg (2018) (eight from one department), Mulli et al. (2022) (11 academics), Smadi et al. (2021) (11 academics) and Jacobs (2016), which involved a single academic response. Table 5 provides summary of findings.

**Table 5.** General study characteristics.

Study characteristics	Count	Percentage
Publication year		
2015–2020	7	63
2021–2024	4	36
Study origin		
United Kingdom	3	27
France, Sweden	2	18
Canada	2	18
France	1	9
South Africa	1	9
Australia	1	9
United States	1	9
Study design		
Qualitative	8	72
Quantitative	1	9
Mixed method	2	18
Data collection technique		
Questionnaire/survey	6	54
In-person interview	3	27
Focus group	2	18
Case study	2	18
Database search	3	27

#### Summary of themes

Four themes were developed from 11 included studies: (1) Reflective practice and the role of academics, (2) Reflective practice and academic challenges, (3) Reflective practice learning approaches and (4) Reflective practice and academic vulnerability.

#### Reflective practice and the role of academics

Academics were found to be vital to promoting reflective learning; they need to balance building trust with challenging students’ ideas. Several tensions were identified in how academics supported reflective learning, including navigating control versus uncertainty and managing closeness while maintaining appropriate distance (Gustavsson et al., 2019). Lindberg (2018) also recognised that academics were supervising students while developing their own reflective capacity. Although reflection was highly valued, many academics doubted their reflective skills and felt uncomfortable modelling it for students, which could undermine the authenticity of reflection in curricula (Jacobs, 2016; Mulli et al., 2022; Naicker and van Rensburg, 2018). Nonetheless, this modelling was seen as essential for demonstrating critical thinking, adaptability and openness (Gustavsson et al., 2019; Jack and Illingworth, 2019).

In three of the 11 studies, academics identified barriers, including poor simulation design, inadequate assessment options and insufficient preparation, that hindered reflective practice. Academics raised concerns that these hindrances stemmed from poor preparation, teaching methods and the selection of, or lack of knowledge about, the reflective tool and framework (Jacobs, 2016; Lindberg, 2018; Mulli et al., 2022). Both Timizar-Le Pen et al. (2020) and Smadi et al. (2021) reported feeling unprepared to design, implement and evaluate reflective assessments, resulting in an ad hoc approach that was often descriptive rather than reflexive, thereby lessening the effectiveness of the intended learning outcomes.

#### Reflective practice and academic challenges

Significant challenges were identified in integrating reflective practice into nursing curricula. Both Gustavsson et al. (2019) and Graham and O’Brien (2020) noted that academics are expected to plan, teach and evaluate reflective practice to meet curriculum outcomes. However, three papers reported that academics consistently lacked formal training in reflective methodologies (Lindberg, 2018; Naicker and van Rensburg, 2018; Smadi et al., 2021), leading to uncertainty about which frameworks and tools to use. Time was another significant barrier, with reflective practice often described as an ‘add-on’ in already crowded curricula (Naicker and van Rensburg, 2018; Timizar-Le Pen et al., 2020). Although some models, such as Koldestam et al.’s (2021) Model for Improvement in Learning Outcomes (MILO) framework, provided clear conceptual scaffolding for reflection, most academics reported inadequate support or mentorship opportunities to sustain reflective teaching practices (Graham and O’Brien, 2020; Smadi et al., 2021). Lindberg (2018) highlighted that a lack of self-trust in reflective practice exacerbated these challenges, leaving academics feeling exposed and under-supported. Three papers also reported that the lack of clear institutional strategies affected feedback processes, and without proper preparation or reflective skills, feedback was more likely to be methodological rather than developmental (Graham and O’Brien, 2020; Timizar-Le Pen et al., 2020; Koldestam et al., 2021).

#### Reflective practice learning approaches

The importance of clear pedagogical frameworks for strengthening academics’ confidence in teaching reflective practice was highlighted in five of the papers. Graham and O’Brien (2020) described how implementing a guided reflective model with dedicated time and group sessions required significant preparation and resource allocation for academics. Jack and Illingworth (2019) noted the challenges of promoting student-centred learning within such frameworks but emphasised that, for reflective practice to succeed, it was essential. Similarly, Mulli et al. (2022) and Timizar-Le Pen et al. (2020) noted the demands placed on simulation facilitators to identify and support reflection-in-action, necessitating strategies such as pre-briefing and prompts to manage learner anxiety and fear, which in turn affect reflective outcomes.

Variability in reflective teaching tools was evident across the literature. Timizar-Le Pen et al.’s (2020) study across 16 French nursing schools found considerable differences in how Situation Analysis (SA) was implemented and assessed, whereas the conceptual literature by Jacobs (2016) suggested that reflective activities are frequently shaped by individual academics’ teaching approaches rather than standardised pedagogical methods.

#### Reflective practice and academic vulnerability

The final theme identified was the experience of vulnerability in facilitating reflective practice. Lindberg (2018) and Gustavsson et al. (2019) explained that academics often struggle to balance closeness and distance in their relationships with students, highlighting the tension between maintaining authority and modelling openness. Reflection often requires demonstration to role model its worth and clinical purpose, but it also gives academics the chance to demonstrate authenticity and build trust (Gustavsson et al., 2019; Mulli et al., 2022). These qualities, in turn, influence both the outcome of teaching and the learning of reflective practice (Jacobs, 2016). Jack and Illingworth (2019) demonstrated how integrating poetry as a reflective tool requires educators to help students adopt unfamiliar, creative methods. Although these approaches call for more guidance and sensitivity from academics, they enable richer empathy and shifts in perspective, highlighting the need for academics to balance vulnerability with pedagogical innovation. Similarly, Smith (2021) found that guided reflective writing offered academics a structured way to scaffold reflection across different student levels, providing security for both students and academics while encouraging deeper critical thinking. These findings demonstrated that academics are expected to navigate vulnerability, adopt alternative methods and create safe spaces for reflection whilst still challenging the students.

## Discussion

This scoping review drew on the perspectives and experiences of academics teaching reflective practice to undergraduate Bachelor of Nursing students. Findings from the included studies highlighted a consistent narrative: although reflective practice is recognised as vital in nursing education, effective implementation remains challenging. Academics frequently report feeling underprepared, under-supported and time-constrained, limiting their ability to integrate reflection meaningfully into teaching (Lindberg, 2018; Mulli et al., 2022; Naicker and van Rensburg, 2018). It is important to note at this time, some studies refer to ‘participants’ as both academics and students.’ For this review, only academics will be included in the discussion.

A central theme across the included studies is the gap between the theoretical value of reflection and its practical application. Many academics, particularly those transitioning from clinical practice into academia, are confident in their professional expertise but lack the pedagogical preparation to facilitate reflective learning (Gustavsson et al., 2019; Lubbe and Botha, 2020). These academics often feel overwhelmed by a lack of clarity about how to teach, assess and model reflection (Bagheri et al., 2019; Graham and O’Brien, 2020). Even when reflective practice is included in curricula, it is not always integrated into teaching culture, and academics are not adequately supported to deliver it effectively (Lubbe and Botha, 2020; Naicker and van Rensburg, 2018).

Several studies emphasise the importance of academics modelling reflection, engaging in their own reflective processes, addressing their vulnerabilities and actively building trust with students (Gustavsson et al., 2019; Mulli et al., 2022; Naicker and van Rensburg, 2018). However, many academics face structural and organisational barriers that hinder this modelling, including inadequate training, poorly designed simulations, limited time and a lack of feedback (Jacobs, 2016; Lindberg, 2018; Mulli et al., 2022). These barriers not only hinder students’ engagement but also diminish academics’ confidence in the value and authenticity of reflective practice.

The literature highlights tensions between authentic reflection and performative, assessment-driven practices, with reflection often reduced to a task rather than supported as a transformative learning process (Clarke, 2021; Edwards, 2017; Rolfe, 2014). Academics also report difficulties adapting reflective activities to diverse student needs, noting that although some students benefit from journaling or one-to-one dialogue, others engage more effectively through group or peer-led approaches (Agnew, 2022; Holdo, 2023). Academics further describe uncertainty about professional boundaries and the emotional labour involved in facilitating reflection, at times feeling overwhelmed by the responsibility of supporting students’ personal and professional growth (Gustavsson et al., 2019; Mulli et al., 2022; Naicker and van Rensburg, 2018). Without appropriate support, reflective practice risks becoming superficial or tokenistic rather than a meaningful pedagogical process (Andersen and Toubøl, 2024; Bagheri et al., 2019; Helberget et al., 2021).

Sherwood (2024) called for a review of reflective practice in higher education that reconnects with its philosophical foundations, promoting curiosity and critical questioning, whereas Agnew (2022) supports academics and students in their growth through reflection. This requires systemic investment in training, mentoring and structural time, as well as a cultural shift within institutions to value reflection as a scholarly and transformative activity (Lubbe and Botha, 2020). In summary, this review highlights the need to provide academics with the time, training and resources to teach reflective practice meaningfully. Educational organisations must prioritise a dual focus: equipping students with reflective capabilities while empowering academics to model, guide and evaluate reflection with integrity and authenticity.

## Implications and Conclusion

This scoping review found limited research on nurse academics’ perspectives and experiences of teaching reflective practice to undergraduate nursing students. Despite its recognised value for developing students’ critical thinking, professional judgement, and theory-practice within undergraduate courses, the implementation of reflective practice remains inconsistent. Academics report insufficient training, institutional support, time, and confidence to apply reflective frameworks, often reducing reflection to a superficial or assessment-driven task rather than a transformative learning process.

Importantly, academics’ vulnerability, preparedness, and authenticity directly shape the depth and quality of the reflective learning they can facilitate. This indicates that improving student outcomes requires investment in academics themselves, not only in curricular frameworks. Educational institutions should therefore prioritise structured pedagogical support, dedicated training, mentoring, and adequate time, enabling academics to model and facilitate reflection with integrity and confidence.

Further research into academics’ lived experiences of managing these personal and institutional demands is needed to inform curriculum development and better prepare future nurses for practice. Without this investment, the transformative potential of reflective practice in nursing education remains unrealised.

## Strengths and limitations

A key strength of this scoping review is the authors’ systematic search strategy across six major databases, supported by clearly defined PCC criteria to capture relevant studies. The review was informed by the JBI methodological framework and quality appraisal tools, which enhanced its rigour and transparency. Data extraction and thematic analysis were conducted collaboratively, enhancing consistency and reducing reviewer bias.

Limitations should be acknowledged. The number of eligible studies was small, and sample sizes in some were limited, which may have restricted the range of perspectives represented. Variability in study designs, contexts and approaches to reflective practice was also evident. Only English-language publications were included, which may have excluded relevant international research. Despite these limitations, this review offers valuable insights into the academic perspectives and experiences of teaching reflective practice in undergraduate Bachelor of Nursing programmes in Australia and internationally and highlights the need for further research to better understand how they manage the personal and institutional demands of teaching reflection. Future research may inform curriculum development, enhance academic growth and better prepare future nurses for the complexities of practice.

**Key points for policy, practice and research**

- Nursing programmes should work towards implementing consistent, institution-wide frameworks and protected time for reflective practice to prevent ad hoc, assessment-only reflective practice implementation.
- Academics require protected time with structured training and mentoring to confidently model, facilitate and assess reflective practice.
- Flexible, inclusive reflective options are needed to meet diverse student learning needs.
- Further research is needed to understand how academics’ reflective capacity, vulnerabilities and professional identities influence the quality of reflective learning in their teaching.
